# Targeting the Hippo Pathway in Cutaneous Melanoma

**DOI:** 10.3390/cells13121062

**Published:** 2024-06-19

**Authors:** Urszula Kazimierczak, Anna Przybyla, Marianna Smielowska, Tomasz Kolenda, Andrzej Mackiewicz

**Affiliations:** 1 Department of Cancer Immunology, Poznan University of Medical Sciences, Rokietnicka Street 8, 61-806 Poznan, Poland; 2 Department of Genome Engineering, The Institute of Bioorganic Chemistry, Polish Academy of Sciences, Z. Noskowskiego 12/14, 61-704 Poznan, Poland; 3 Laboratory of Cancer Genetics, Greater Poland Cancer Centre, Garbary 15, 61-866 Poznan, Poland; 4 Research and Implementation Unit, Greater Poland Cancer Centre, Garbary 15, 61-866 Poznan, Poland; 5 Department of Cancer Diagnostics and Immunology, Greater Poland Cancer Centre, Garbary Street 15, 61-866 Poznan, Poland

**Keywords:** cutaneous melanoma, Hippo pathway, therapeutic targets

## Abstract

Melanoma is the most aggressive form of skin cancer. In the advanced stage of development, it is resistant to currently available therapeutic modalities. Increased invasiveness and metastatic potential depend on several proteins involved in various signal transduction pathways. Hippo signaling plays a vital role in malignant transformation. Dysfunctions of the Hippo pathway initiate the expression of tumor growth factors and are associated with tumor growth and metastasis formation. This review summarizes the recent achievements in studying the role of the Hippo pathway in melanoma pathogenesis and points to the potential specific targets for anti-melanoma therapy.

## 1. Introduction

Melanoma is among the most aggressive human cancers. In a metastatic phase, it is resistant to the conventional treatment. Clinically, it is a highly heterogeneous disease, which may translate into systemic therapy failures. Despite recent progress in the identification of several therapeutic targets or inhibitors of immune checkpoints, metastatic melanoma is still incurable. So far, little is known about its pathogenesis, and the specific biomarkers that would allow for a better diagnosis and prediction of the disease course are missing. The high invasiveness and metastatic potential of melanoma result from mutations and the activation of different signal transduction pathways. The Hippo signaling controls tissue and organs’ growth and differentiation. It is also largely involved in tumor formation and metastasis. However, the specific mechanisms of how the Hippo pathway controls melanoma are unknown. Characterizing the critical proteins involved in major signaling pathways and analyzing their interactions will allow for a better understanding of the mechanisms of melanoma pathogenesis and the identification of its specific clinical subtypes. That will allow the identification of specific molecular targets for more effective treatment in the future.

## 2. The Hippo Pathway

Hippo signaling was initially discovered in *Drosophila melanogaster*. It is an evolutionarily conserved pathway that controls organ growth during the development of all metazoans [1,2]. The primary role of the Hippo pathway is the thorough supervision and regulation of organ size, cell proliferation, apoptosis, and stemness in response to intrinsic and extrinsic signals [3]. Dysregulation of the pathway results in organ and tissue hypertrophy and is associated with a neoplastic phenotype and cancer metastasis [4].

## 3. Main Components

**Figure 1:**
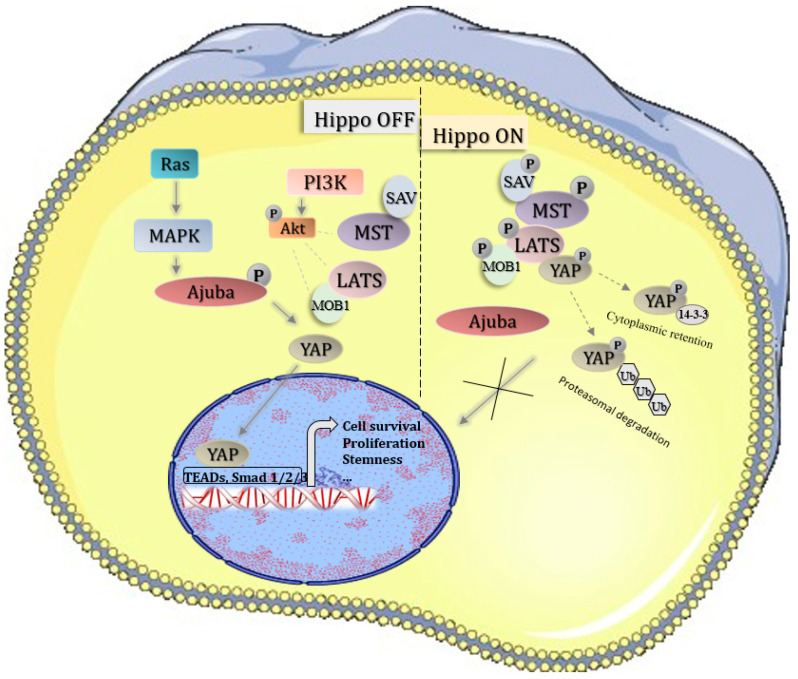
Hippo signaling pathway; inactive Hippo leads to YAP oncogene transport to the nucleus and activation of pro-survival genes; active Hippo leads to YAP phosphorylation and its degradation in the cytoplasm.

The human Hippo pathway comprises three interlinked parts, namely, the upstream regulatory components, the core kinase cascade, and the downstream transcriptional machinery. The core of the Hippo pathway acts rather as a signaling network than as a definite transduction cascade [5]. It contains a conserved kinase cassette consisting of MST1/2, LATS1/2, and MOBKL1A/B and MOBKL1B (MOB1) kinases [6,7]. MST1/2 (Mammalian Ste20-like kinase 1/2) forms a complex with SAV1 (*Salvador* Family WW Domain Containing Protein 1), which phosphorylates and activates the kinase complex composed of LATS1/2 (Large tumor suppressor 1/2) and MOB1 (*MOB* Kinase Activator) [8]. While activated, LATS1/2 phosphorylates and inactivates YAP1 (Yes-associated protein 1) and its paralog TAZ (also known as WWTR1) [8,9]. Unphosphorylated YAP1 and TAZ easily enter the nucleus and function as transcriptional co-activators. However, while phosphorylated, YAP1 and TAZ are bound by 14-3-3 proteins, consequently sequestered in the cytosol, and degraded by proteasome complexes [10,11]. The schematic representation of the Hippo signaling pathway is shown in Figure 1.

## 4. Upstream Regulators of the Core Kinase Cascade

Hippo signaling has been linked to a diverse array of upstream signals, including cell polarity, adherens junctions, the cytoskeleton, mechanical forces, GPCR ligands, and cellular stress [12]. The first identified upstream components of the Hippo pathway in *Drosophila* were two FERM-domain-containing proteins: Merlin (NF2 in mammals) and Expanded (FRMD6 in mammals) [13]. NF2 is the product of the Neurofibromatosis type 2 (NF2) tumor suppressor gene and a member of the ezrin–radixin–moesin (ERM) protein family [14,15]. The carboxy-terminal domain (CTD) is joined with the N-terminal FERM domain in its closed inactive conformation. Phosphorylation of C-terminal residues disrupts the connection and liberates FERM to bind cell adhesion molecules. NF2 functions in a complex with WWC1 (Kibra) and FRMD6 as an upstream effector of the Hippo pathway in order to regulate cell growth and suppress tumor development. The WWC1-NF2-FMRD6 protein complex activates the MST1 protein through autophosphorylation [14,16]. WWC1 is an apical protein containing two amino-terminal WW domains, an internal C2-like domain, and a carboxy-terminal glutamic-acid-rich region. To promote Hippo signaling, NF2 binds to Kibra (WWC1 in mammals). Another upstream regulator of the Hippo pathway is the transmembrane protein Crumbs (Crb), which functions in a complex with FRMD6 [13]. The Hippo pathway can be also regulated by cell–cell junction proteins such as e-cadherin or alpha-catenin [17,18]. The increased adherence and tight junctions in confluent cells contribute to the activation of LATS and the inactivation of YAP/TAZ [19]. Detachment of the cells inactivates YAP/TAZ and triggers anoikis [20]. At low cell density, LATS kinase remains inactive [10]. Cell–cell contact also influences YAP activity by altering cell geometry. Cells grown on stiff ECM or spread across a large surface are flat and show nuclear localization of YAP, while cells grown on soft ECM, on a small surface, or detached from a culture plate are round, and YAP is localized in the cytoplasm [21]. The Hippo pathway responds to different forms of cellular stress that cause energy deficits. It plays a role in oxidative stress [22], ER stress [23], osmotic stress [24], cytokinesis failure [25], and pathogens [26,27]. A prominent role in Hippo signaling regulation is played by striatin-interacting phosphatase and kinase (STRIPAK), which inactivates MST1/2 through its dephosphorylation [28].

## 5. The Core of the Hippo Signaling Pathway

### 5.1. Mammalian Ste20-like Kinases (MSTs) 1/2

Mammalian Ste20-like kinase (MST1/2) is classified among the STE20 kinases. Primarily, MST1/2 was described as an elemental proapoptotic kinase that is activated through signals linked to stress-induced apoptosis [29,30]. The activity of MST1/2 is modulated both by autophosphorylation and caspase cleavage [31]. MST1/2 activity is also regulated by heterodimerization with RASSF family tumor suppressors. In human cancers, the expression of RASSF proteins is frequently restricted through epigenetic silencing [32]. Conversely, following caspase cleavage, modified MST protein with a liberated N-terminal kinase domain enters the nucleus and induces chromatin condensation by phosphorylating histone H2B, causing DNA fragmentation and apoptosis [8,33]. MST1/2 activity is also regulated by heterodimerization with RASSF family proteins, which are tumor suppressors. In human cancers, the expression of RASSF proteins is frequently restricted through epigenetic silencing [32]. Experiments performed on mice proved that MST1/2 proteins are essential for proper development. A single knock-out of one MST1 or MST2 allele does not lead to any disorders. However, mice that have both alleles knocked out die in embryonal stadiums due to developmental defects [34].

### 5.2. Large Tumor Suppressors (LATSs) 1/2

LATS1 and LATS2 belong to the serine–threonine kinase family and are involved in cell proliferation, apoptosis, and cell cycle regulation. They are mainly active in the early prophase, as LATS1 cooperates with CDC2/cyclin A and LATS2 binds to the proteins in the centrosome region, enabling the formation of a kinetic spindle. Additionally, LATS1 negatively regulates the G2/M transition by downregulating CDK1 kinase activity [35]. Correspondingly, LATS2 inhibits cell growth at the G1/S control point by downregulating cyclin E/CDK2 kinase activity [36]. Regarding the Hippo pathway, LATS1 and LATS2 are tumor suppressors that inhibit the oncogenic nuclear function of YAP/TAZ and TEAD. Following phosphorylation by MST1/2, LATS1/2 connects with the coactivator MOB1 and undergoes autophosphorylation. Simultaneously, SAV plays an essential role as a scaffold protein for assembling MST1/2 and LATS1/2 [37,38]. Consequently, this active LATS1/2-MOB1 complex phosphorylates the transcriptional coactivators YAP and TAZ on their multiple HxRxxS motifs, leading to their inactivation [39].

### 5.3. Mps One Binder Kinase Activator (MOB) 1

MOB 1 is an essential coactivator of LATS kinases. Overexpression of MOB1 triggers LATS1/2 activation and consequently leads to proliferation inhibition or even to apoptosis. Contrarily, a decrease in MOB1 expression increases the rate of cell division. MOB1 deficiency or reduction is frequently associated with tumorigenesis, especially in human skin melanoma, colorectal cancer, and non-small-cell lung cancer [40].

## 6. Hippo Pathway Effectors

### 6.1. Yes-Associated Protein (YAP) and WW-Domain-Containing Transcription Regulator 1 (WWTR1/TAZ)

YAP and TAZ are the effector proteins in the Hippo pathway. They are paralogs with 46% amino acid identity. As they do not have a DNA-binding domain, YAP and TAZ are classified as transcriptional coactivators, which interact with transcriptional factors in order to regulate gene expression [41]. Although YAP and TAZ are mostly considered functionally redundant, some evidence supports their differential roles in organ morphogenesis and cancer development and progression. The common structural feature of YAP and TAZ is the WW domain. The WW domain is a protein–protein interaction domain with two conservative tryptophan residues. Different isoforms of YAP and TAZ may differ in the number of WW domains. YAP1 and TAZ have a single WW domain, whereas YAP2 bears a double WW domain [42]. The presence of one or two WW domains may influence the set of YAP and TAZ interactors [43]. YAP1 and YAP2 contain also an SH3-domain-binding motif and PDZ-binding motif in their C-terminus [44]. YAP/TAZ activity depends on their cellular localization, since only while being in the cell nucleus may they exert their functions. Contrarily, following the phosphorylation of a conserved serine residue (S127 on YAP and S89 on TAZ), they bind with 14-3-3 protein and, therefore, become inactivated and restrained outside the nucleus. Apart from serine 127, there are four other consensus HXRXXS motifs that may be phosphorylated on YAP (S61, S109, S164, and S381). Nonetheless, S127 and S381 are regarded as critical sites related to the oncogenic phenotype. Phosphorylation of solely S127 suffices to impede YAP activity [45]. When phosphorylated at S127, YAP is sequestrated in the cytoplasm via interaction with 14-3-3 protein [46]. Phosphorylation of S381 on YAP and S311 on TAZ leads to YAP/TAZ polyubiquitination and degradation [43,47]. YAP and TAZ perform the role of an oncogene in medulloblastoma, glioblastoma multiforme, squamous cell carcinoma, small-cell lung cancer, liver cancer, colorectal cancer, pancreatic cancer, and ovarian cancer [48]. Noteworthily, YAP overexpression leads to neoplastic transformation as cells gain the ability to proliferate infinitely and uncontrollably, to undergo the epithelial–mesenchymal transition (EMT), and to evade apoptosis [49]. Additionally, YAP and TAZ make cancer cells evade immune surveillance [50]. Adversely, while in most solid tumors, YAP is an oncogene, in hematological cancers, it plays (unlike TAZ) a tumor-suppressive role [51].

### 6.2. TEA Domain (TEAD) Factors

TEAD belongs to a family of transcriptional factors that mediate the main transcriptional output of the Hippo pathway in response to YAP and TAZ activity [52,53]. All members of the TEAD family contain an identical DNA-binding domain that binds the M-CAT motif (5′-CATTCCT-3′) in the genes that they regulate [54]. TEAD transcription factors are expressed in every kind of tissue. The levels of particular TEAD factors vary, and their physiological response may differ depending on the tissue type. For example, the YAP protein is indispensable for the full transcriptional activity of TEAD2. Interaction between TEAD1–4 and their major binding partners, YAP and TAZ, causes the dissociation of VGLL4 from TEAD1–4 and the anchorage of YAP/TAZ to chromatin. Consequently, TEAD-mediated gene transcription induces cell proliferation and inhibits apoptosis [55].

## 7. The Hippo Pathway in Cancer

**Figure 2:**
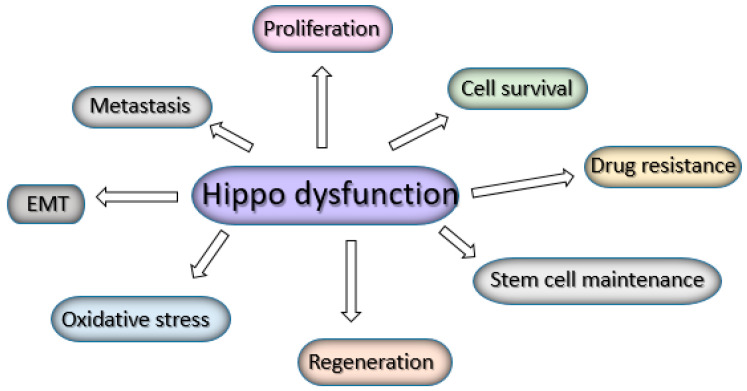
Hippo pathway dysfunction leads to several events linked with cancer progression.

The Hippo signaling pathway is dysregulated in a variety of cancers, leading to YAP/TAZ activation, which, in turn, leads to the hyperproliferation of cancer cells, a lack of apoptosis and stem cell maintenance, the epithelial-to-mesenchymal transition and increased invasion, metastatic potential, oxidative stress, and, finally, resistance to therapies [8,56] (Figure 2).

**Figure 3:**
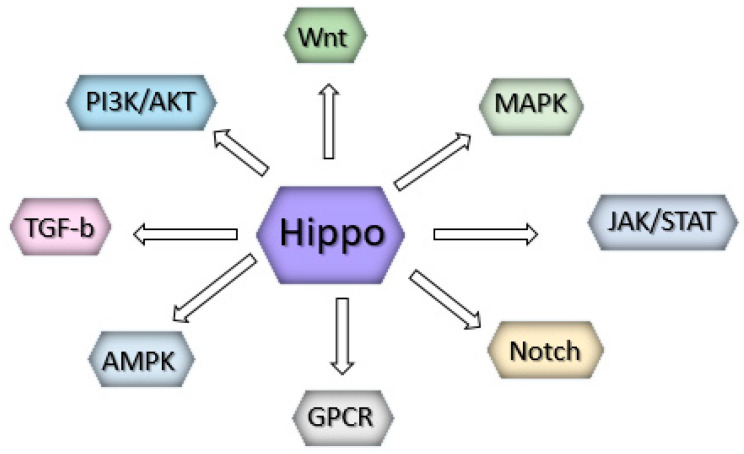
Hippo signaling highly impacts several pathways involved in melanoma development.

However, these events result from mutual cooperation with other prominent signaling pathways, such as the Wnt, G-protein-coupled receptor (GPCR), epidermal growth factor (EGF), BMP/transforming growth factor beta (TGFβ), JAK/STAT, and Notch pathways [57,58]. In melanoma, the interaction of the Hippo pathway with the PI3K/Akt (Akt) and ERK/Raf/Ras (MAPK) pathways is essential for tumor growth control [59] (Figure 3).

## 8. The Hippo Pathway in the Biology and Treatment of Melanoma

Components of the Hippo pathway are involved in the determination of the overall number of cutaneous melanocytes. Mechanical signals are mediated by junctions between melanocytes and the basement membrane, and other melanocytes and keratinocytes are integrated in space and time to activate the Hippo signaling pathway as it takes place in other tissues. These signals are complemented by biochemical markers sent from keratinocytes and transferred from surface receptors to cellular components through protein phosphorylation [60]. Clinical data confirmed that the deregulation of the Hippo pathway is often correlated with poor prognosis for melanoma patients [61].

### 8.1. Hippo Signaling and BRAF/MEK Inhibitor Resistance

Targeted therapy with BRAF and MEK inhibitors significantly improved the prognosis of melanoma patients. However, this still demonstrates some limitations [62,63]. Most patients treated with BRAF inhibitors acquire resistance within 1 or 2 years. Defining the mechanisms of that phenomenon is an emerging issue that needs to be addressed to improve the outcomes. It has been previously shown that YAP promotes resistance to anti-cancer therapies. In the past decade, the YAP mechanotransduction pathway has been implicated in the resistance to BRAF and MEK inhibitors. Understanding the mechanisms leading to YAP-related resistance to RAF and MEK inhibitors is essential to develop a more effective treatment. Lin et al. confirmed the unanticipated functional cross-talk between YAP and RAF-MEK signaling. The authors investigated whether YAP regulates the response to the targeted inhibition of BRAF signaling in several tumor cell lines harboring BRAF-, KRAS-, or NRAS-activating mutations [64]. They found that YAP1 suppression enhanced the response to vemurafenib and trametinib in A2058 and WM793 melanoma cell lines and A2058 melanoma xenografts. Most likely, YAP enhances the expression of antiapoptotic factors such as BCL2 family member protein BCL-xL in resistant cells, and YAP knockdown prevents that process. Moreover, YAP upregulation in BRAF- or RAS-mutant tumor cells might promote resistance to RAF and MEK inhibitors in patients. Accordingly, the authors observed high YAP levels in most melanoma samples with mutant BRAF V600E. Interestingly, patients harboring BRAF V600E mutation who responded to RAF and MEK inhibitors (CR) exhibited lower YAP expression in the pretreatment tumor samples, and those with incomplete responses had higher YAP expression before treatment [64]. The above study clearly shows that YAP acts as a survival factor in melanoma cells, and its downregulation may be an effective way to enhance the therapeutic effects of RAF- and MEK-inhibitor-based treatment.

Searching more profoundly into the mechanisms of BRAFi resistance, Kim et al. looked at the connection between the Hippo pathway and the anti-melanoma immune response. They genetically modified three melanoma cell lines (SKMEL28, WM3248, and A375) to activate YAP and performed a set of in vitro experiments to test whether YAP activation changes the cytokine release by those cells and how it influences the cytotoxic abilities of lymphocytes. They found that YAP activation in BRAFi-resistant melanoma cells directly inhibited cytotoxic T-cell responses, and the mechanism that they proposed was the induction of PD-L1 expression on tumor cells [65,66].

Kim et al. studied the resistance mechanism to the BRAF inhibitor PLX4032 in melanoma cell lines and linked it with actin cytoskeleton remodeling. They demonstrated that melanoma cells resistant to PLX4032 exhibit increased stress fibers in the actin cytoskeleton, which appears to promote the nuclear accumulation of YAP/TAZ [67]. After knocking down YAP in PLX4032-resistant SK-MEL28 and WM3248 cell lines, they observed the reduced viability of the cells, whereas overexpression of constitutively active YAP induced resistance of the parental melanoma cell lines. YAP/TAZ knockdown also influenced EGFR and phosphorylated AKT levels and resulted in significant enrichment of downregulated genes related to the cell cycle and mitosis in PLX4032-resistant cells. Activated YAP overexpression suppressed the expression of SOX10 and MITF and upregulated EGFR. The authors also showed that both the protein and mRNA levels of c-MYC depend on YAP/TAZ. Conversely, inhibition of actin polymerization and actomyosin tension suppresses YAP/TAZ activation and PLX4032 resistance. The results of the above study suggest that the inhibition of actin remodeling may be a potential strategy to suppress the resistance to BRAF-inhibitor-based therapies [67]. Also, Monaghan-Benson found a connection between the Hippo pathway and BRAF-related modification of the cell cytoskeleton. Mutated BRAF was associated with a Rac-dependent cadherin switch in melanoma cells. Rac1 acts to modify the cytoskeleton. This gene is highly mutated in melanoma cells, and loss of its function can change the regulation of the Hippo pathway [68].

Recently, Dieter et al. described a novel approach for breaking YAP-/TAZ-driven resistance to MAPK pathway inhibitor treatment. First, they transiently activated YAP1 in BRAFV600E mutant melanoma cell lines and established that it is YAP1 that induces the resistance to BRAF and MEK inhibitors. Moreover, YAP1 was shown to drive the MITFlow/AXLhigh phenotype, which seems to be a potential biomarker predicting response to therapeutic approaches using MAPKi. Next, using a genome-wide CRISPR/Cas9 functional screen, the authors identified the essential genes in MAPKi-resistant cells with activated YAP1. They distinguished the *SLC35B2* gene, which appeared to be crucial in that process. SLC35B2 is responsible for heparan sulfation expression and positively correlates with tumor progression. After suppressing the gene, they noticed that this modification sensitized the cells to BRAF inhibition [69].

BRAF inhibitor resistance can also be triggered by the ubiquitin ligase-dependent degradation of LATS1 and MST2 in BRAFi-resistant cell lines. Romano et al. demonstrated that mutant BRAFV600E binds to and inhibits MST2, preventing MST2-dependent apoptosis. Treatment of BRAFi-resistant cells with proteasome inhibitors resulted in the rescue of proapoptotic MST2 and LATS1 signaling. The results from a small cohort of patients with resistance to BRAFi confirm that MST2 downregulation might be associated with the acquisition of resistance in human melanoma [70].

### 8.2. YAP/TAZ in Melanoma Invasion and Metastasis

Several studies have discussed a connection between the Hippo signaling targets and melanoma pathogenesis. Nallet-Staub et al. provided evidence that endogenous YAP/TAZ contributes to melanoma cells’ invasiveness and metastatic behavior. They performed profiling of human melanocytic lesions and melanoma cell lines for endogenous YAP and TAZ expression, followed by specific YAP/TAZ knockdown and overexpression experiments in melanoma cell lines to analyze the anchorage-independent growth, invasion into Matrigel, human skin reconstruction, and in vivo tumor growth and metastasis formation. They found that the knockdown of either of these Hippo effectors led to reduced clonogenic and invasive capacity in vitro and decreased their ability to form lung metastases after intravenous injection in mice. In contrast, YAP/TAZ overexpression had the opposite effects. Interestingly, TAZ levels were higher than YAP in melanoma cells, and its knockdown gave a better anti-melanoma response than YAP knockdown. The authors of the above study noticed that overexpression of YAP and TAZ leads to enhanced nuclear activity despite their phosphorylated forms in the cytoplasm. YAP/TAZ overexpression increased anchorage-independent growth and invasion into Matrigel, confirming a direct role for endogenous YAP and TAZ in controlling melanoma cells’ metastatic potential [71]. This study corroborates the findings of Lamar et al. from 2012, as they showed that overexpression of YAP enhanced the frequency and size of melanoma tumors in lungs after intra-vein injection in a TEAD-binding-dependent manner. They used the A375 melanoma cell line for Luminex-based in vivo assays. They found that the domain of YAP that interacts with the TEAD/TEF family of transcription factors is essential for tumor cell proliferation, transformation, migration, and invasion. They also observed that the ability of melanoma cells to form metastases is significantly correlated with increased TEAD transcriptional activity [72]. They generated an activated YAP by introducing the S127A mutation into YAP and found that it dramatically enhanced mouse tumor growth and metastasis. Mutation of the TEAD interaction domain prevented tumor formation [72]. In conclusion, YAP-mediated melanoma growth and metastasis seem to require a functional TEAD interaction domain. In addition, YAP exerts its prometastatic effects at both the primary and metastatic sites, enhancing several processes that contribute to metastasis.

### 8.3. YAP/TAZ and CSC Phenotype

A group of scientists from the University of Maryland School of Medicine searched for the role of YAP/TAZ in cancer stem cells (CSCs). They studied BRAFi-resistant melanoma stem cell spheroids (MCSs) and noticed their enhanced growth, Matrigel invasion, and elevated YAP1, TAZ, and TEAD levels. The knockdown of TEAD proteins reversed that effect. They also performed knockdown and overexpression experiments to confirm the BRAFi resistance role of YAP1 and TAZ, since inhibiting YAP1 function with verteporfin reduced the YAP1/TAZ levels and restored sensitivity to BRAFi [73]. YAP1 expression can be downregulated after treatment with the combination of the histone deacetylase inhibitor LBH589 and the bromodomain inhibitor I-BET151, as was shown in melanoma xenografts. Both drugs induced caspase-dependent apoptosis, which, except for the YAP1 decrease, involved the downregulation of the AKT pathway [74].

### 8.4. YAP/TAZ as Prognostic Factors

Menzel et al. used the comparative genomic hybridization (CGH) data of 48 human primary melanomas and ten melanoma metastases to analyze the involvement of the YAP1 gene in melanoma development. They found that in twenty tumors, the YAP1 gene or YAP1-activating PAK1 gene was amplificated or there was a copy number loss of LATS2 or NF2 suppressor genes. Also, patients with high YAP1 expression showed decreased survival [75].

YAP1 activity has been shown to be especially elevated in constitutively invasive melanoma cells. Zhang et al. demonstrated that YAP1 hyperactivity could switch melanoma cells from proliferative to invasive phenotypes and promote spontaneous melanoma metastasis in vivo while compromising the growth of primary tumors. Moreover, that switch cannot be reversed, and in consequence, such invasive cells either die or downregulate YAP1 overexpression to increase the chance of survival [76].

Zhang et al. depleted YAP and/or TAZ in several melanoma cell lines and used patient-derived xenografts (PDXs) to test the therapeutic effects of YAP targeting. They discovered that the sensitivity of YAP1 or TAZ targeting was independent of classical BRAF/NRAS driver mutations; however, it was variable between different cell lines or patients. They also showed that YAP1 is elevated in most melanomas and may be considered a survival factor for melanoma cells. On the other side, the YAP1 level was also increased in benign nevi, the vast majority of which did not transform into an invasive state. From that point of view, YAP1 does not seem to be a good prognostic biomarker for YAP1-targeted therapy, and other marker proteins need to be elucidated [77].

Yuan et al. analyzed different genetic variants (single-nucleotide polymorphisms) of the Hippo pathway genes in melanoma patients to check whether they were associated with survival rates. The authors demonstrated that *YAP1*rs11225163 C>T, *TEAD1* rs7944031 A>G, and *TEAD4* rs1990330 C>A significantly modulated the survival rates of melanoma patients. Moreover, based on genotyping data, they could distinguish different prognostic groups. This study confirmed the importance of combining genetic features with clinicopathological characteristics while assessing a patient’s prognosis [38].

### 8.5. YAP/TAZ Signaling Regulation

The evidence that YAP overexpression is an essential factor for tumor progression and resistance to targeted drugs is apparent. However, little is known about the regulatory mechanisms of YAP/TAZ signaling. Studies have shown that non-coding RNAs (ncRNAs) can directly or indirectly regulate it. Choe et al. demonstrated that miR-550a-3-5p directly suppressed oncogenic YAP and exerted tumor-suppressive activity. Treatment of vemurafenib-resistant melanoma cells with miR-550a-3-5p reduced the phosphorylation of AKT, marginally modulated phosphorylated ERK, and, consequently, improved the sensitivity to the drug. The authors also observed that the resistant WM3248 melanoma cell line showed a higher level of EGFR and maintained ERK/AKT activity in response to vemurafenib compared with control cells. miR-550a-3-5p overexpression inhibited various oncogenic properties, including cell proliferation, anti-apoptosis, migration, invasion, and cancer stemness, consistently with YAP inhibition effects [78].

Hippo signaling regulation is essential for melanoma development. Kim et al. indicated that the PIN1-STK3 axis might regulate the transcription of TAZ/TEAD target genes in melanoma cells. PIN1 is one of the factors that inhibit the Hippo pathway. It is associated with STK3 (MST2) and promotes its ubiquitin-mediated degradation, which induces the nuclear translocation of the TAZ oncogene and subsequent transcriptional activation of the TAZ/TEAD complex. TAZ/TEAD enhances the transcription of specific genes, such as *CTGF*, which promotes cell proliferation, epithelial–mesenchymal transition, invasion, and cellular transformation [79,80]. Conversely, depletion of PIN1 in melanoma cells leads to retention of TAZ in the cytoplasm, followed by a decrease in CTGF and the final inhibition of melanoma development. The authors demonstrated that PIN1 increases the mRNA and protein expression of TAZ/TEAD target genes in A375 cells, promoting tumorigenicity, and STK3 overexpression suppressed the tumorigenicity of those cells. These results were further confirmed by in vivo experiments with a syngeneic mouse model. Moreover, TCGA analysis of SKCM patients revealed a significant negative correlation between PIN1 and STK3 mRNA levels [81].

While studying the mechanisms of melanoma pathogenesis, researchers from China focused on the role of the latent transforming growth factor binding protein (LTBP). They previously demonstrated that LTBP4 may serve as a diagnostic marker for melanoma in the GSE46517 dataset [82]. Patients with low expression of that gene had poorer prognosis. It has been shown that LTBP4 stabilizes the TGF-β receptor complex and regulates TGF-β1 activity, while TGF-β1 regulates the nuclear translocation of YAP1 protein [83]. Thus, LTBP4 regulates the Hippo pathway by affecting TGF-β1 activity. With his group, Wang silenced LTBP4 in melanoma cells and observed the enrichment of YAP1 in the nucleus and the inhibition of phosphorylation of YAP1 protein in the cytoplasm. Thus, they indicated that LTBP4 promotes the phosphorylation of YAP1 and reduces its nuclear translocation, leading to the activation of the Hippo signaling pathway [84].

## 9. The Core of the Hippo Pathway in Melanoma Suppression

### 9.1. LATS1/2 in Tumor Growth Regulation

Although most studies on the Hippo signaling pathway’s engagement in melanoma are focused on the YAP1 oncotarget, several pieces of evidence point to the high importance of its core kinases in regulating that cancer. The Hippo pathway consists of four tumor suppressors: SAV1, MST1/2, LATS1/2, and MOB kinase activators. The role of the upstream kinases is to degrade their oncotargets (YAP/TAZ) and inhibit melanoma growth and metastasis. SAV1 is involved early in the Hippo pathway and is required to activate LATS1/2. Moreover, SAV1 directly interacts with AKT kinase, suppressing AKT-mediated tumorigenesis and promoting apoptosis. It is clear that SAV1 and LATS1/2 play essential roles in tumor suppression. Romano et al. demonstrated that SAV1 and LATS1/2 expression can be induced by triptonide. Triptonide is a minor component in the Chinese herb Tripterygium wilfordii and exerts a strong anti-cancer effect with low toxicity. Enhanced expression of SAV1 and LATS1/2 leads to oncogenic YAP degradation and suppression of MITF and AKT. These events strongly inhibit melanoma cell tumorigenicity, migration, invasion, and lung metastasis [85].

Our previous study showed that LATS1 silencing reduces the expression of the melanogenesis markers and melanin synthesis in primary melanocytes and melanoma cells. That phenomenon was associated with increased LATS1 knocked-down human xenograft growth in nude mice. Moreover, silencing LATS1 resulted in enhanced oxidative stress [22].

Vittoria et al. studied controlled BRAFV600E expression in a melanocyte cell line and discovered that the expression of oncogenic BRAFV600E leads to a significant increase in LATS phosphorylation and, consequently, the nuclear exclusion of YAP, as well as a decrease in the expression of the YAP target genes CYR61 and AMOTL2. Also, it restrains oncogenic melanocyte proliferation in vitro due to the activation of the Hippo pathway. Next, the authors searched for the activation mechanism and found that since inhibition of MEK1/2 or ERK1/2 kinases resulted in YAP/TAZ phosphorylation, the Hippo pathway’s activation is entirely mediated by the general hyperactivation of MAPK signaling. In vivo experiments showed that the functional impairment of the Hippo pathway in melanocytes promoted cutaneous melanomagenesis. They showed that Lats1/2−/− mice formed tumors with strong p-ERK staining, suggesting that LATS1/2 depleted melanocytes evolved to hyperactivate the MAPK pathway. In addition to highlighting the role of LATS1/2 kinases in melanoma development, that study also pointed to YAP/TAZ as promising therapeutic targets, as constitutively active YAP caused extended tumor formation in zebrafish [61]. In 2016, Moroishi et al. published a prominent paper that describes the suppressing role of LATS1/2 in melanoma immunity. They discovered that inactivation of LATS1/2 in tumor cells strongly suppresses tumor growth in immune-competent but not immune-compromised mice, which is caused by the induction of anti-tumor immune responses. LATS1/2-deficient melanomas secreted nucleic-acid-rich extracellular vesicles (EVs) that stimulated the host TLR-MYD88/TRIF-IFN nucleic-acid-sensing pathways, inducing anti-tumor inflammatory reactions. Using three different melanoma cell lines in different syngeneic mouse models, they showed that LATS1/2 deletion abolished the growth of SCC7 tumors and highly reduced tumor growth and the metastasis of B16 and 4T1 cells [86].

It has been demonstrated that LATS1 kinase directly links the Hippo pathway with apoptosis. Garcia-Gutierrez et al. performed a set of experiments to identify new interactions of LATS1 with apoptotic markers. They found that LATS1 forms complexes with SMAC, which depends on RASSF1A expression. SMAC is a mitochondrial protein that promotes cytochrome-c-dependent caspase activation. The LATS1–SMAC complex requires the presence of the IAP family member XIAP. Although XIAP inhibits SMAC alone, it seems that LATS1 bound to SMAC counteracts that effect, resulting in increased SMAC levels due to protein stabilization. SMAC then promotes the ubiquitination and subsequent degradation of XIAP, leading to apoptosis. In a parallel study, the authors observed the loss of MST2 and LATS1 expression in BRAFi-resistant melanoma cell lines. They showed that oncogenic BRAF can inhibit MST1/2 in these cell lines and prevent MST2–LATS1 interactions and LATS1-dependent apoptosis [87].

### 9.2. LATS and microRNA in Melanoma Tumorigenesis

Hu et al., using an online tool for target prediction analysis of microRNA (miRDB), linked LATS2 kinase with miR-135b expression and showed its role in melanoma tumorigenesis. The authors compared miR-135b levels in 20 melanoma tissues and analyzed the impact of miR-135b on cell proliferation, migration, or apoptosis in either primary melanocytes or the A-375 melanoma cell line. MiR-135b expression was significantly upregulated in melanoma tissue. Overexpression of miR-135b in primary melanocytes promoted cell proliferation and migration, and miR-135b inhibition reversed that effect. The proposed mechanism of such a process was that miR135b, by targeting LATS2, prevents its inhibitory effects [88].

Surprisingly, LATS1 phosphorylation of YAP1 may also indirectly promote the growth of a small population of cancer cells named tumor-repopulating cells (TRCs). TRCs were discovered by Jiu et al., who mechanically selected a small subpopulation of cancer cells with high tumorigenic and proliferative capacity and named them tumor-repopulating cells (TRCs). Those cells were shown efficiently to repopulate tumors at distant organs in mice and were independent of surface CSC markers. Using microarrays, the authors screened for potential factors contributing to enhanced proliferation of TRCs and identified that Nuclear protein 1 (Nupr1) was markedly reduced in that population. YAP1 phosphorylation by LATS1 leads to the downregulation of Nupr1, leading to the downregulation of p53 and upregulation of Nestin and Tert. All of these events help to promote the proliferation and self-renewal of TRCs [89].

### 9.3. MST1/2 Activation and Suppression of Tumor Growth

The efficacy of the Hippo pathway is significantly dependent on Merlin, which is an upstream regulator of MST1/2 Ser/Thr kinases and responds to the inhibition of cell proliferation and invasiveness. Murray et al. assessed the correlation between Merlin expression and response to oxidative stress. They found that higher Merlin levels in human melanoma cells promote the H_2_O_2_-induced activation of MST1/2 and the suppression of tumor growth. Merlin knockdown promoted post-confluence cell proliferation and subcutaneous growth of WM1552C human melanoma cells [90].

The efficacy of the Hippo pathway is significantly dependent on Merlin, an upstream regulator of MST1/2 Ser/Thr kinases, and it responds to the inhibition of cell proliferation and invasiveness. Murray et al. assessed the correlation between Merlin expression and response to oxidative stress. They found that higher Merlin levels in human melanoma cells promote the H_2_O_2_-induced activation of MST1/2 and suppression of tumor growth. Merlin knockdown promoted post-confluence cell proliferation and subcutaneous growth of WM1552C human melanoma cells. Feng et al. analyzed the interactions between the MAPK and Hippo pathways in four melanoma cell lines. They found that RAF-1 forms a complex with MST-2. The suppression of RAF-1 prevents RAF-1/MST-2 complex formation, leading to MST-2 activation and the subsequent inhibition of cell proliferation, migration, and invasion, as well as apoptosis promotion. These results agree with another study on interactions between different signaling pathways. Notably, Romano et al. reported that RAF-1 mutation stimulates both the Hippo and MAPK pathways, simultaneously driving apoptosis and proliferation, whereas concomitant MST-2 downregulation switches signaling to cell proliferation, transformation, and survival [91].

Yu et al. linked the Hippo pathway with curcumin-induced melanoma cell apoptosis. They found that curcumin activated MST1-dependent cell death in cultured melanoma cells. Curcumin enhances ROS production, which activates MST1. Activated MST1 promotes cell apoptosis by inducing JNK activation, Foxo3a nuclear accumulation, and Bim-1 expression. MST1 knockdown prevented curcumin-induced activation of caspase-3, caspase-9, and Bim expression, contributing to cancer cell apoptosis [92].

Kang et al. studied a link between the Hippo pathway and fascin in melanoma. Fascin is upregulated in various cancers, including melanoma. It increases melanoma tumorigenesis and stemness via interaction with MST2 and the inhibition of MST2 homodimer formation and kinase activity, thus reducing LATS activity and enhancing TAZ (but not YAP1) stability. The results point to the fascin/Hippo axis as a potential therapeutic target for melanoma. Knockout of fascin significantly reduces the tumorigenicity and stemness of melanoma, whereas the ectopic expression of fascin exerts opposite effects. Thus, targeting fascin could also become an effective strategy for advanced melanoma [93].

## 10. Summary

The Hippo pathway has been investigated in tumorigenesis due to its particular role in cell growth and metastasis. It affects several biological processes, such as cell proliferation, migration, invasion, epithelial-to-mesenchymal transition, cancer stem cell formation, apoptosis, cell cycle, and polarity. The Hippo pathway’s effector components are often mutated or downregulated in various cancers, including melanoma, and the target genes are upregulated. Therapies targeted to the Hippo pathway’s components may largely improve the survival rates of melanoma patients. The thorough analysis of the Hippo pathway in melanoma and the investigation of its relationship with other key signaling pathways involved in tumor formation will provide a better understanding of the mechanisms of melanoma pathogenesis. This will help in the development of more effective treatments.
